# Histological Effects of Intra-Testicular Injections of Cadmium Chloride in Domestic Fowl

**DOI:** 10.1038/bjc.1964.29

**Published:** 1964-06

**Authors:** J. Guthrie

## Abstract

**Images:**


### HISTOLOGICAL EFFECTS OF INTRA-TESTICULAR INJECTIONS OF CADMIUM CHLORIDE IN DOMESTIC FOWL

J. GUTHRIE

*From the Department of Pathology, St. Mary's Hospital, London, W.2*

Received for publication June 9, 1964

THE carcinogenic action of solutions of zinc salts when injected directly into the avian testis was first established by Michalowsky (1926, 1928 and 1929) who reported the production of teratomas. Later Falin and Anissimowa (1940) described similar effects of copper salts. Although the exact mechanism of this action at cellular or subcellular level is still obscure, the teratoid tumours induced by this procedure arise close to the site of inoculation and adjacent to the zone of haemorrhagic necrosis produced (Guthrie, 1964). Chemically, it should be remembered that zinc (atomic No. 30) and copper (atomic No. 29), closely related transition elements in the periodic table, readily form complex ions or chelates with organic molecules. They also play a part in enzyme structure. Pařízek and Záhŏr (1956) reported on the destructive effects of cadmium on the rodent testis following its subcutaneous injection. Cadmium (atomic No. 48) is in the same group as zinc and copper, and six years later Haddow, Dukes, Roe and Mitchley (1962) and Heath, Daniel, Dingle and Webb (1962) added cadmium to the list of carcinogenic metals. Following their work it seemed desirable to assess the effects of local direct injection of a solution of cadmium salts into the avian testis.

### MATERIALS AND METHODS

Analytical grade cadmium chloride ($CdCl_2$) was dissolved in distilled water B.P. to make a 2 g./100 ml. solution. This had a pH of 3·6.

Thirty-eight White Leghorn cockerels 11 months old received bilateral intra-testicular injections of this 2 per cent solution in March 1963, using the intercostal approach previously described (Guthrie, 1964). The birds were kept on a dry mash and mixed corn diet in semi-open verandahs and were killed at intervals varying from 5 days to 7 weeks after inoculation. Immediately after death, the roof was removed from the skull and the pituitary gland exposed by removal of the surrounding bone by bone forceps. Dissection was completed after 24 hours fixation in 10 per cent formalin and the brain and attached pituitary gland then bisected in the sagittal plane. Testes and other organs taken for examination were similarly fixed and all material was dehydrated, cleared and embedded in paraffin. Sections of the testes were stained with haematoxylin and eosin (H. & E.) and Perls' method for iron. The pituitary gland was stained H. & E., by periodic acid-Schiff method (PAS) and Gomöri's aldehyde fuchsin.

### RESULTS

### *Effects on the testis*

Immediately following the injection slight blanching of the area around the injection site was noticed. In a few cases there was subcapsular haemorrhage, mainly due to injury of the subcapsular veins, but in general the effects were less severe than those which followed the inoculation of zinc chloride. Nevertheless all the inoculated fowl showed localised testicular lesions. The principal findings are tabulated (Table I).

TABLE I.—*Results of Bilateral Intra-testicular Injection of 2 per cent $CdCl_2$ Solution in 38 White Leghorn Cockerels*

| | | Lesions in testes | | | | | | |
|---|---|---|---|---|---|---|---|---|
| Ref. No. of bird | Length of experiment (days) | Right or left testis | Scar | Necrosis | Pigment | Sperm granuloma | Other | Gonadotrophic activity of adenohypophysis.† |
| B142 | 5 | Both | .. | + | .. | .. | Haemorrhage | No definite departure from controls. |
| Y187 | 18 | Both | .. | + | + | .. | Dilated tubules, tubules lined only by Sertoli cells | No definite departure from controls. |
| 4197, B141 | 30 | Both | .. | + | + | .. | Haemorrhage, dilated tubules | Increase. |
| B139 | 45 | Both | .. | + | .. | .. | | |
| B143 | 45 | Both | + | .. | + | .. | Horn cyst in right | |
| B147 | 45 | Both | + | .. | + | .. | | Marked increase. |
| B150 | 45 | Both | + | + | + | .. | | |
| 339 | 47 | Right | + | + | .. | + | | Increase. |
| | | Left | + | + | .. | .. | Haemorrhage | |
| 343 | 47 | Both | + | .. | + | .. | Haemorrhage and horn cyst + mucin in left | |
| 341* | 47 | Both | + | + | + | .. | | |
| 353 | 47 | Both | + | .. | + | .. | | |
| 309, 313, 321 | 48 | Both | + | .. | + | .. | | |
| 315 | 48 | Both | + | .. | .. | + | | |
| 323, 327 | 48 | Both | + | + | .. | .. | | |
| 329, 333 | 49 | Both | + | + | + | .. | Sertoli cell adenomas in right | Marked increase. |
| 331, 335, 337 | 49 | Both | + | + | .. | .. | | |
| 347 | 49 | Right | + | .. | + | .. | | |
| | | Left | + | + | + | .. | Dilated tubules | |
| 349 | 49 | Both | + | .. | + | .. | Dilated tubules | |
| 351 | 49 | Both | + | + | .. | .. | | |
| 357 | 49 | Both | + | .. | + | .. | | |
| 307 | 50 | Both | + | .. | + | + | | |
| 311 | 50 | Both | + | + | + | .. | | |
| 319 | 50 | Both | + | .. | .. | + | | |
| 335, 345 | 50 | Both | + | .. | .. | .. | | |
| 317, 325 | 51 | Both | + | + | .. | + | | |
| B140, 301 | 52 | Both | + | + | + | .. | | |
| 303 | 52 | Both | + | + | .. | .. | | |
| 305 | 53 | Both | + | .. | + | .. | Teratoma in right, including horn cysts | Increase. |

* No. 341 showed depressed spermatogenesis ; all other birds showed the active spermatogenesis of early summer.

† Examined in 9 birds.

The cockerel, No. 142, killed 5 days after inoculation showed a gross lesion rather similar to that following zinc chloride. Histologically there was a zone of coagulative necrosis involving seminiferous tubules and interstitium including the walls of blood vessels. The appearances were similar to those produced by zinc chloride and as with zinc the ghost outlines of spermatogenic cells, several with pyknotic nuclei, were retained in these necrotic areas 7 weeks after inoculation (Fig. 1). This case shows the dense infiltration by lymphocytes and occasional multinuclear giant cells around the remains of the necrotic tubules and in the scar the regeneration of Leydig cell groups as well as the presence of the darker pigment containing cells. In and close to the scar of the inoculation, sperm granulomas of varying size were found. These showed curious regimentation of the spermatozoa, especially at their peripheries, and were surrounded by a lymphocytic and histiocytic reaction (Fig. 2). The largest of these were about 2 mm. in diameter and were clearly visible to the naked eye as whitish nodules arranged around the pigmented scar or necrosis.

Immediately outside the scar and mainly under the capsule there was a variable number of tubules lined only by Sertoli cells, but beyond this spermatogenesis appeared normal for the season in the intact seminiferous tubules.

In two cases, No. 329 and 333, the cadmium induced scar was surrounded by several solid white nodules of 3 to 4 mm. in diameter, the superficial nodules projecting from the surface of the testis. Histologically these consisted of tubules, devoid of spermatogenic cells, in which the Sertoli cells had proliferated sufficiently to justify the designation of Sertoli cell adenomas (Fig. 3).

Pigmented macrophages were present in the scars in 22 birds. A large proportion of the pigment gave the Prussian blue reaction for iron : some gave a positive reaction for haematoidin. Small horn cysts were found in the scars in 3 cases and in one of these, No. 343, microcysts containing mucin and keratin with transitions between squamous and mucin secreting cubical epithelium were seen (Fig. 4).

One fowl, No. 305, killed 53 days after inoculation, showed in the right testis, close to the scar, horn cysts, nodules of cartilage, adipose tissue, smooth muscle bundles and complex glandular structures with papillary infoldings (Fig. 5, 6, 7 and 8). The appearances were those of a well differentiated teratoma.

*Effects on the adenohypophysis*

The brains and attached pituitary glands of 9 cockerels with cadmium induced lesions and 3 normal cockerels of the same age were available for examination.

The 3 normal fowl and 7 of the cadmium injected fowl whose pituitary glands were obtained were killed at the same time in early May, i.e. 7 weeks after the intra-testicular injections of cadmium chloride. The other 2 were killed at 5 days and 18 days after injection.

Histologically the avian adenohypophysis shows considerable variation with season and environmental conditions. Recent reports on its structure are those of Mikami (1958) and Tixier-Vidal (1962). With the exception of the pituitary glands of the cockerels killed 5 and 18 days after inoculation all the pituitary glands of the cadmium injected cockerels showed increased numbers of PAS positive cells in cephalic and caudal lobes of the pars distalis when compared with the normal controls. A proportion of these PAS positive cells contained large PAS positive vacuoles which in some cases coalesced to displace the nucleus in a signet ring fashion. There was also a variable number of PAS positive granules lying extra-cellularly in places. The large intra-cellular vacuoles and the vast majority of the cells with PAS positive granules in the caudal lobe did not stain with Gomöri's aldehyde fuchsin.

In the 2 birds with Sertoli cell nodules, No. 329 and 333, these changes were more marked.

*Effect on other organs*

No gross lesions were observed in the other organs. In 4 fowl histologically examined, no abnormalities were noted in liver, kidneys, lungs, heart and adrenal glands.

### DISCUSSION

Attributing a carcinogenic action on an endocrine organ to a substance does not mean that the effect is necessarily a direct one on the tissue forming the neoplasm. For example, damage to the seminiferous epithelium, the target tissue of the gonadotrophic and other hormones, is likely to lead to increased gonadotrophic activity if the damage is extensive enough. This increased gonadotrophic activity may then lead to hyperplasia and neoplasia of testicular tissue. The Leydig cell growths produced by Haddow *et al.* (1962) by injection of cadmium sulphate could be associated with increased output of interstitial cell stimulating or luteinizing hormone and thus be in the same category as those produced by implanting testis into the spleen (Jones, 1955) and other procedures associated with degeneration of the seminiferous tubules (Guthrie, 1956). In a somewhat similar category are the Sertoli cell adenomas of the present series. These bear a very close resemblance histologically to the better differentiated canine Sertoli cell

---

### EXPLANATION OF PLATES

FIG. 1.—No. 333. Histological appearances of testis 7 weeks after intra-testicular injection of 2 per cent $CdCl_2$. Note at the top the necrotic seminiferous tubules surrounded by lymphocytes; in the centre, fibrosis with inflammatory infiltrate and Leydig cell groups. At the bottom, a slightly subfertile seminiferous tubule is seen. H. & E. $\times 72$.

FIG. 2.—No. 319. Sperm granuloma associated with ruptured seminiferous tubule in $CdCl_2$ induced scar. The regimented spermatozoa at the top are surrounded by a lymphocytic and histiocytic reaction. H. & E. $\times 195$.

FIG. 3.—No. 333. Edge of Sertoli cell adenoma at the top half of the photomicrograph. Note normal seminiferous tubules at the bottom. H. & E. $\times 73$.

FIG. 4.—No. 343. $CdCl_2$ induced testicular scar showing microcysts containing mucin and keratin with transitions between squamous and mucin secreting cubical epithelium. H. & E. $\times 74$.

FIG. 5.—No. 305. Well differentiated teratoma in $CdCl_2$ scar. Somewhat atrophic seminiferous tubules are seen at top and bottom. The central area shows a group of feather follicles, a horn cyst and a nodule of cartilage with surrounding adipose tissue. The air sac lining is seen on the left. H. & E. $\times 40$.

FIG. 6.—No. 305. Higher magnification of section shown in Fig. 5. Note the group of developing feather follicles at the top, the epidermoid structure with surrounding mesenchyme at bottom left, and the nodule of hyaline cartilage at bottom right. H. & E. $\times 72$.

FIG. 7.—No. 305. Teratoma testis showing complex glandular structure surrounded by smooth muscle fibres at the top, adipose tissue centrally, and atrophic seminiferous tubules in scar at bottom right. H. &. E. $\times 75$.

FIG. 8.—No. 309. Enlargement of glandular structure seen in Fig. 7. Note villous structure bearing columnar cells, mainly ciliated and with a number containing vacuoles giving a positive reaction for mucin. H. & E. $\times 465$.

1 2 3 4

Guthrie.

5 6 7 8

Guthrie.

tumours described by Mulligan (1949), Cotchin (1960) and Dow (1962). They probably take origin in a segment of tubule removed from the site of maximum damage, where the concentration of cadmium is less, for example those isolated segments of tubules lined solely by Sertoli cells seen in the vicinity of the cadmium induced scars in the present series. Cameron and Foster (1963) recorded this tubular appearance in a rabbit 17 days after the subcutaneous injection of cadmium sulphate and also the presence of a neoplastic nodule in the rete testis of another rabbit.

Studies with $^{65}$zinc have shown that after an inoculation of 0·2 ml. of 5 per cent $^{65}$zinc chloride solution into the centre of the testis of the adult rooster, low concentrations of zinc reach either pole (Guthrie, 1962, and unpublished data). Outside the zone of complete necrosis similar selective survival of Sertoli cells was observed with zinc chloride. If a similar diffusion occurred with cadmium chloride, as could be expected from a closely related metal, it would seem that the Sertoli cells are more resistant than the spermatogenic series. Apart from the direct action of the metallic salt on the cells, destruction of whole segments of seminiferous tubules and impairment of blood supply may be a factor in the selective degeneration of the spermatogenic cells.

As a nurse cell, the Sertoli cell is generally accepted to be the homologue of the granulosa cell of the ovarian follicle and thus under the control of the follicle stimulating hormone of the adenohypophysis. It should be noted that both birds with Sertoli cell nodules showed in their adenohypophyses definite increase in PAS positive granularity, a probable indication of increased follicle stimulating hormone secretion. Allanson and Deanesly (1962) found the hypophyseal gonadotrophs hyperactive in the early stages of cadmium treatment in the rat.

Teratoma testis is very rare as a spontaneous growth in the fowl (Mashar, 1932 ; Campbell, 1951) and the finding of an early teratoma in the cadmium induced scar at 53 days after injection points to a causal association rather than a chance finding. The presence of partial castration changes in the adenohypophysis in fowl with established cadmium induced scars is not unexpected, but is in contrast to the absence of these changes in fowl with zinc induced lesions (Guthrie, 1964). The explanation may lie in more extensive damage in the cadmium injected testes.

### SUMMARY

Out of a total of 38 White Leghorn cockerels which received bilateral intra-testicular injections of 2 per cent cadmium chloride solution, one showed an early well differentiated teratoma and 2 others showed multiple Sertoli cell adenomas, all related to the sites of injection.

The pituitary glands of the fowl with cadmium lesions in their testes merely showed changes compatible with partial castration. These changes were more definite in the 2 fowl with multiple Sertoli cell adenomas. The possible role of increased gonadotrophic activity in the induction of these and other non-germinal tumours is discussed.

This work was supported by the British Empire Cancer Campaign for Research. My thanks are due to Mr. E. E. Shorney, Farm Manager at the Park Prewett Hospital, Basingstoke, for his help, and to the Park Prewett Group Management Committee and Dr. I. Atkin, Physician Superintendent, for accommodation.

### REFERENCES

Allanson, M. and Deanesly, R.—(1962) *J. Endocrin.*, **24**, 453.
Cameron, E. and Foster, C. L.—(1963) *J. Anat., Lond.*, **97**, 269.
Campbell, J. G.—(1951) *Brit. J. Cancer*, **5**, 69.
Cotchin, E.—(1960) *J. comp. Path.*, **70**, 232.
Dow, C.—(1962) *Ibid.*, **72**, 247.
Falin, L. I. and Anissimowa, V.—(1940) *Bull. Biol. Méd. exp. U.R.S.S.*, **9**, 518.
Guthrie, J.—(1956) *Brit. J. Cancer*, **10**, 134.—(1962) *Rep. Brit. Emp. Cancer Campgn*, **40**, 290.—(1964) *Brit. J. Cancer*, **18**, 130.
Haddow, A., Dukes, C. E., Roe, F. J. C. and Mitchley, B. C. V.—(1962) *Rep. Brit. Emp. Cancer Campgn*, **40**, 34.
Heath, J. C., Daniel, M. R., Dingle, J. T. and Webb, M.—(1962) *Nature, Lond.*, **193**, 592.
Jones, A.—(1955) *Brit. J. Cancer*, **9**, 640.
Mashar, V.—(1932) *Virchow's Arch.*, **285**, 155.
Michalowsky, I.—(1926) *Zbl. allg. Path. path. Anat.*, **38**, 585.—(1928) *Virchow's Arch.*, **267**, 27.—(1929) *Ibid.*, **274**, 319.
Mikami, S. I.—(1958) *J. Fac. Agric., Iwate Univ.*, **3**, 473.
Mulligan, R. M.—(1949) 'Neoplasms of the Dog.' Baltimore (Williams and Wilkins).
Pařízek, J. and Záhŏr, Z.—(1956) *Nature, Lond.*, **177**, 1036.
Tixier-Vidal, A.—(1962) *Biol. méd., Paris*, **51**, 183.


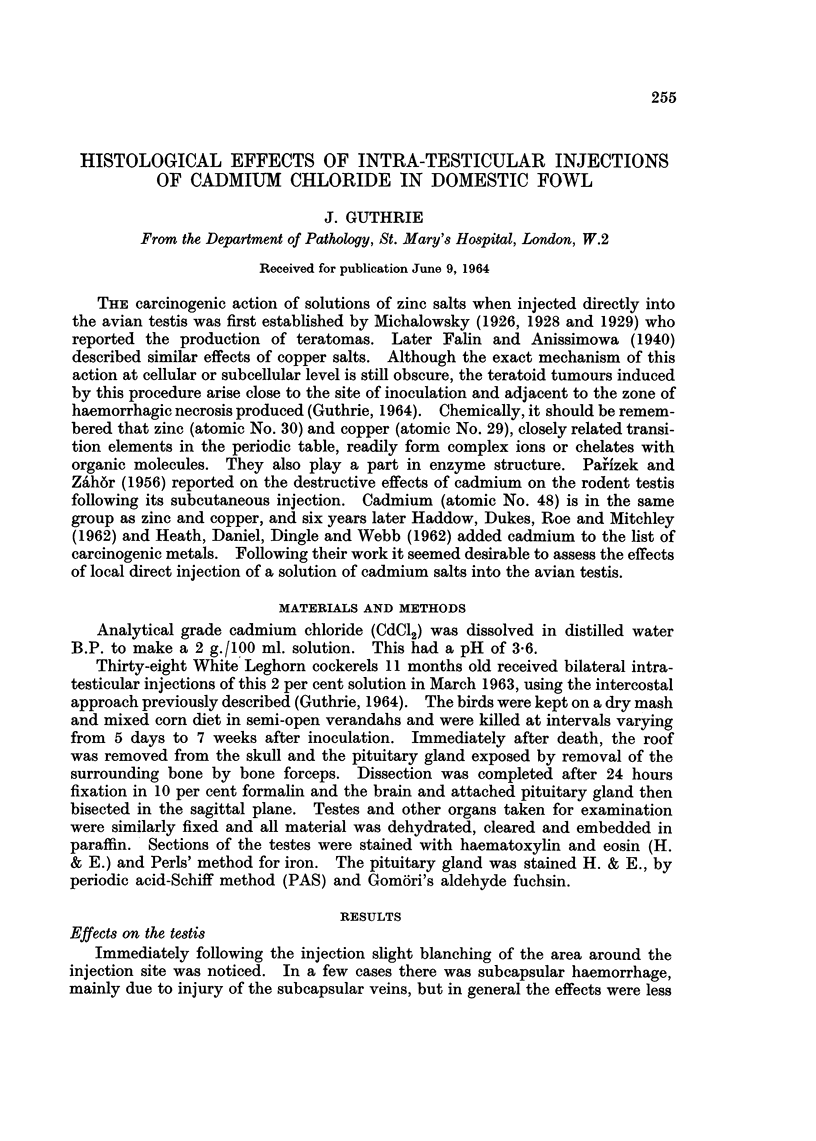

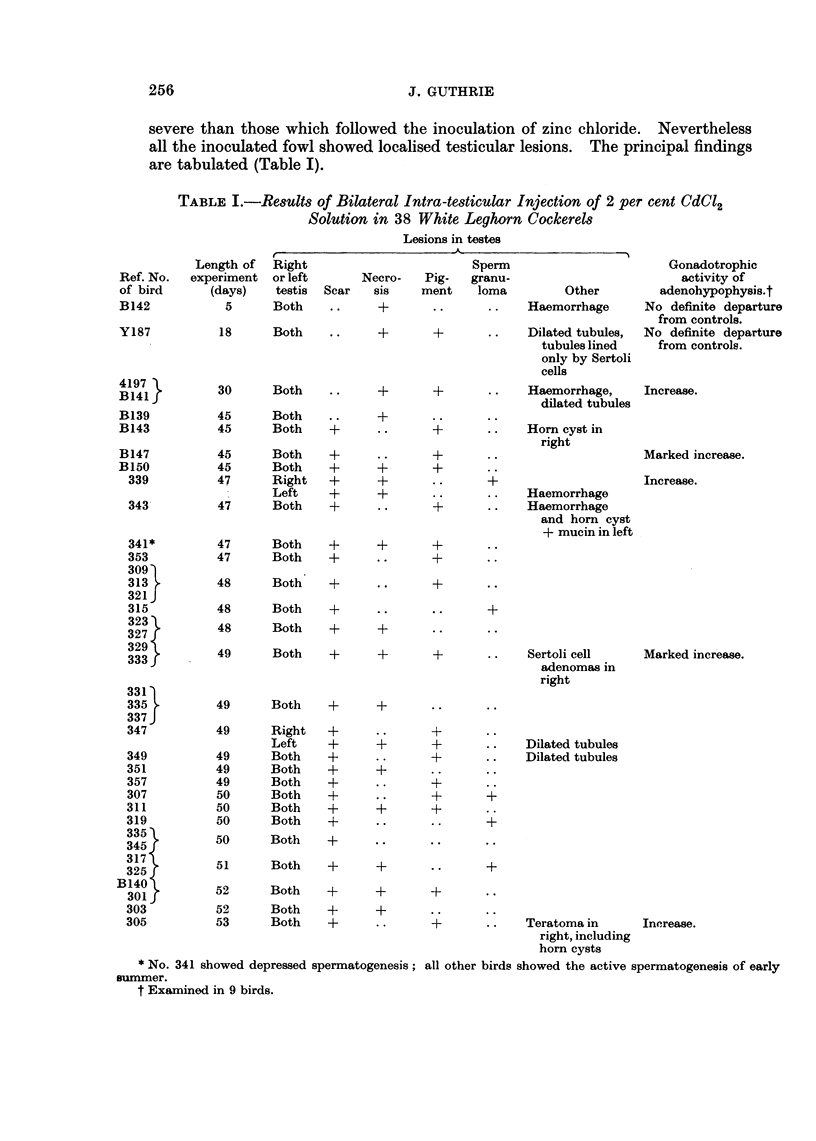

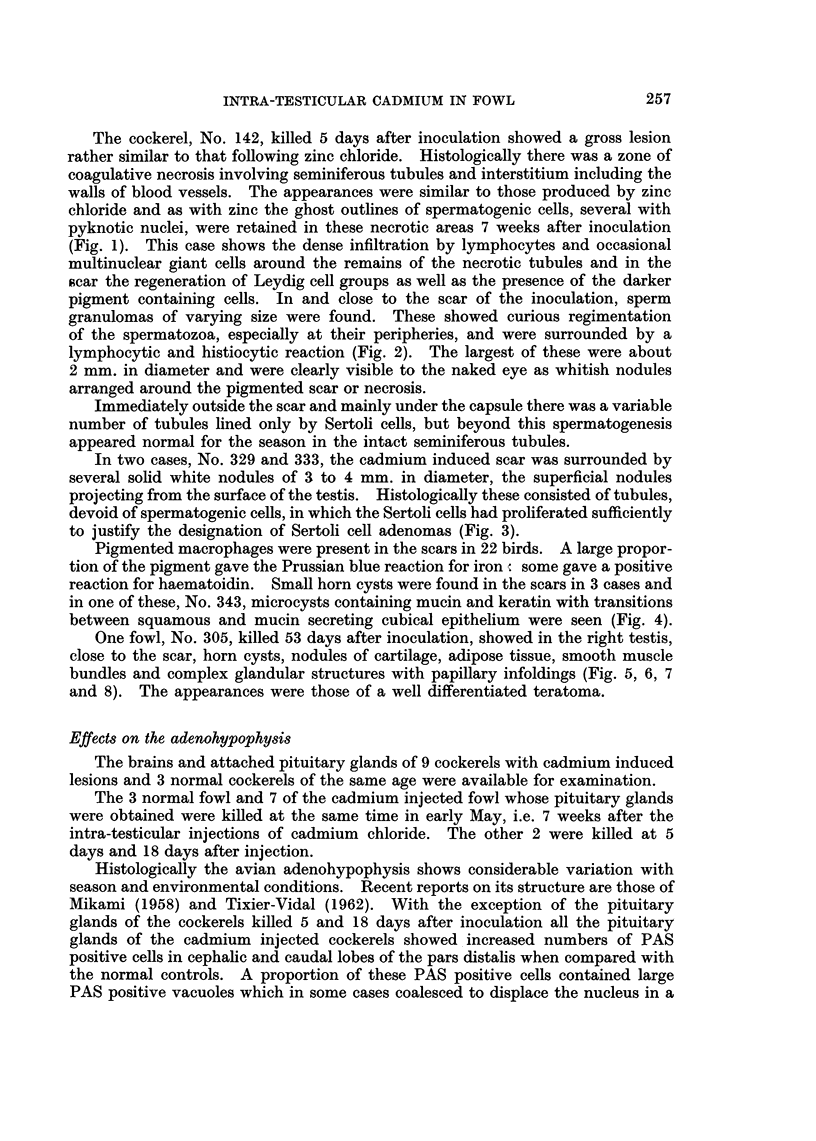

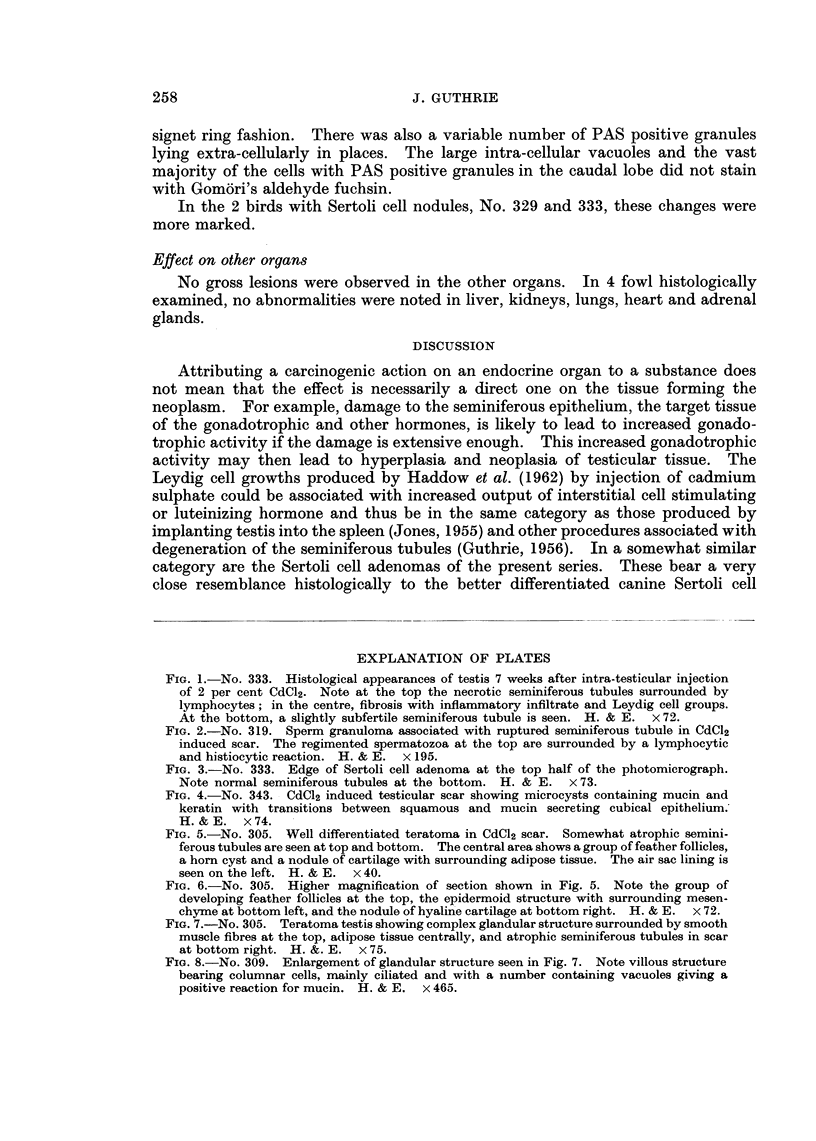

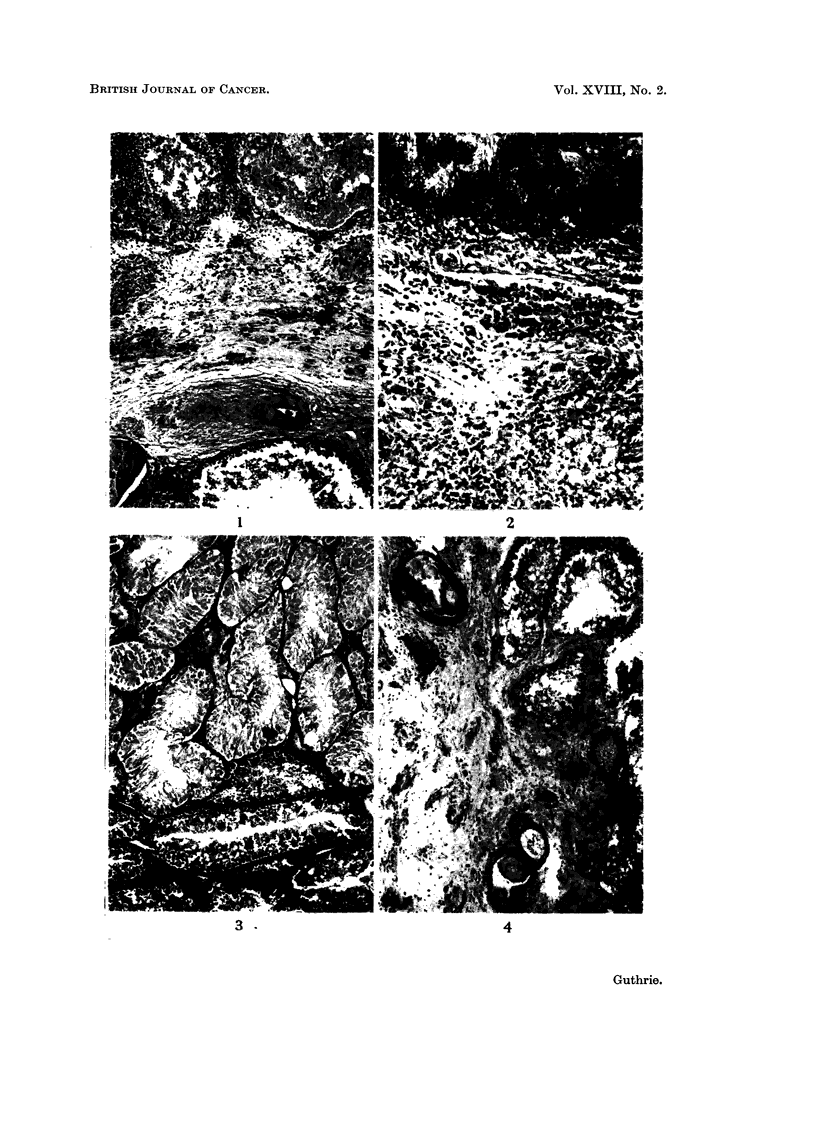

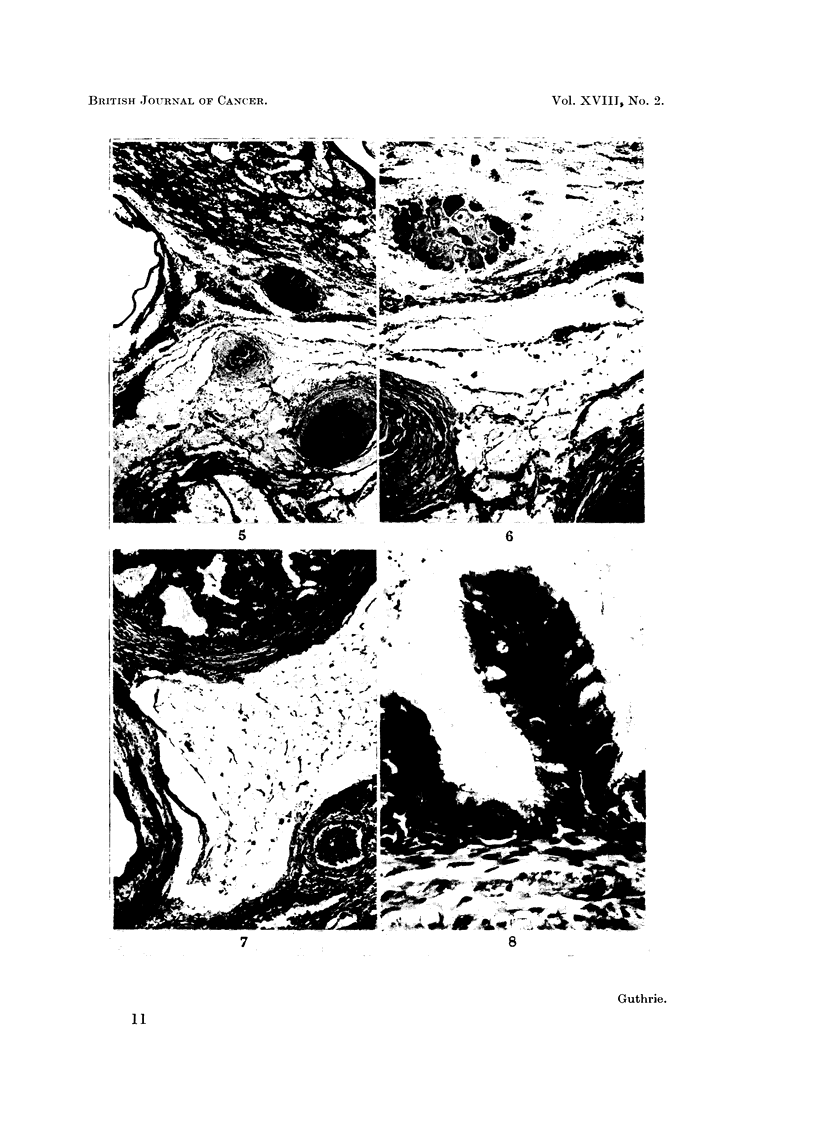

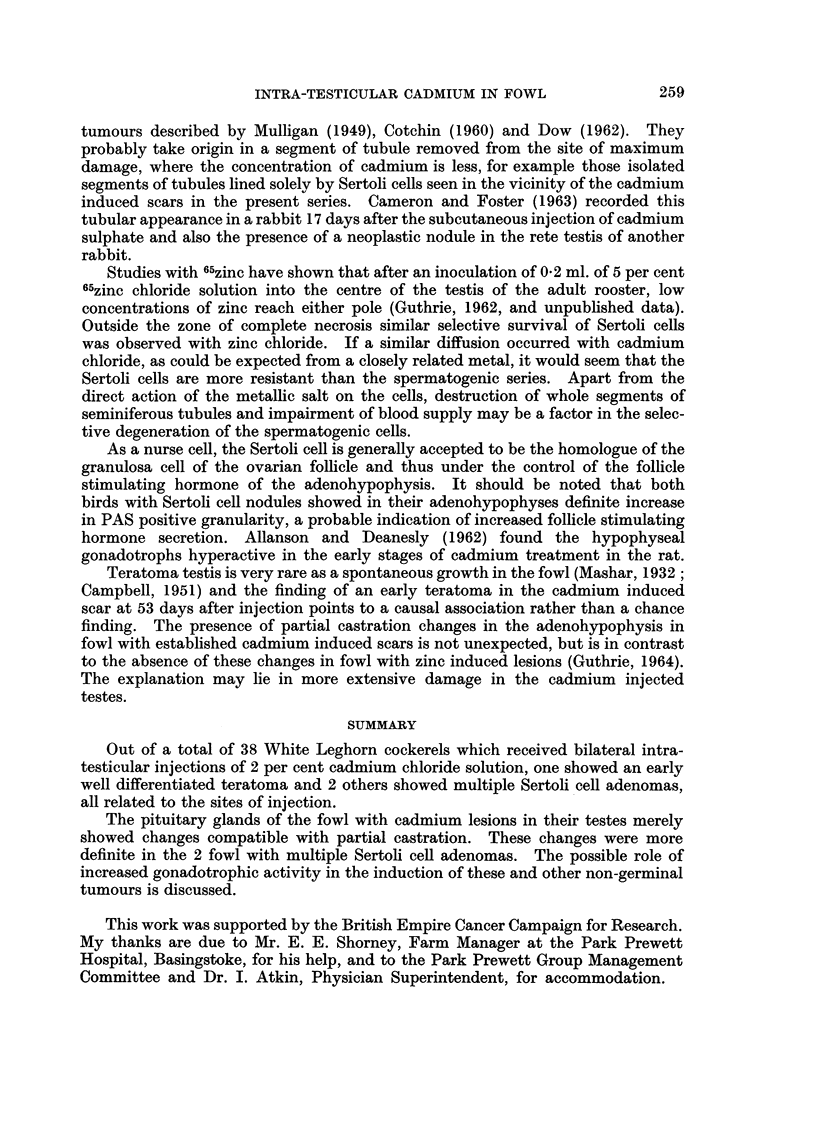

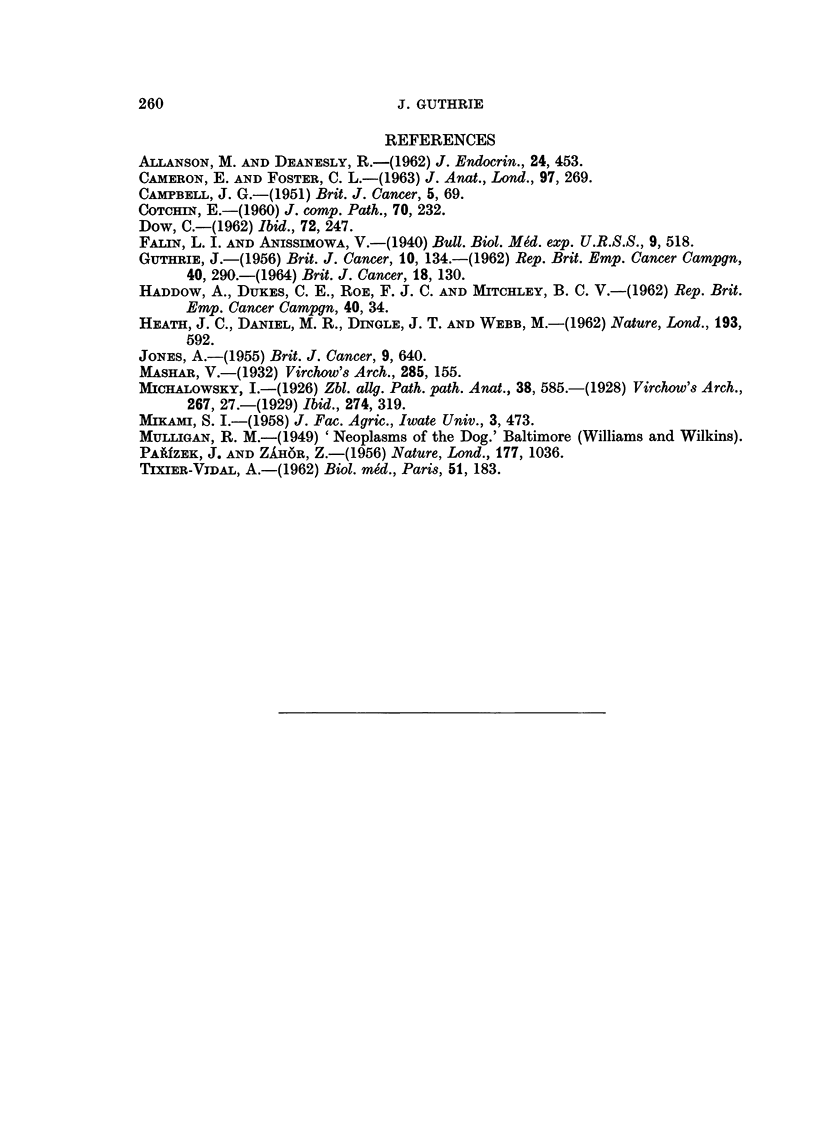


## References

[OCR_00496] CAMERON E., FOSTER C. L. (1963). Observations on the histological effects of sub-lethal doses of cadmium chloride in the rabbit. I. The effect on the testis.. J Anat.

[OCR_00498] DOW C. (1962). Testicular tumours in the dog.. J Comp Pathol.

[OCR_00525] PARIZEK J., ZAHOR Z. (1956). Effect of cadmium salts on testicular tissue.. Nature.

